# MUC21: a new target for tumor treatment

**DOI:** 10.3389/fonc.2024.1410761

**Published:** 2024-06-12

**Authors:** Miao Li, Hui Li, Ting Yuan, Zhi Liu, Yukun Li, Yingzheng Tan, Yunzhu Long

**Affiliations:** ^1^Jishou University Zhuzhou Clinical College, Medical College, Jishou University, Zhuzhou, Hunan, China; ^2^Medical College, Jishou University, Jishou, Xiangxi Tujia and Miao Autonomous Prefecture, Hunan, China; ^3^Department of Infectious Disease, Zhuzhou Central Hospital, Xiangya Hospital Zhuzhou Central South University, Central South University, Zhuzhou, Hunan, China; ^4^Department of Assisted Reproductive Centre, Zhuzhou Central Hospital, Xiangya Hospital Zhuzhou Central South University, Central South University, Zhuzhou, Hunan, China

**Keywords:** MUC21, cancer, infection, biomarkers, immune infiltration

## Abstract

MUC21, also known as Epiglycanin, is a high-molecular-weight glycoprotein with transmembrane mucin properties. It consists of a tandem repeat domain, a stem domain, a transmembrane domain and a cytoplasmic tail. MUC21 is expressed is observed in normal tissues in organs like the thymus, testes, lungs, and large intestine. Research has shown that MUC21 is expressed in esophageal squamous cell carcinoma, lung adenocarcinoma, glioblastoma, thyroid cancer, melanoma, and various other malignant tumors in distinctive manner. Additionally, tumor invasion, metastasis, and poor prognosis are linked to it. Some researchers believe that MUC21 has the potential to become a new target in cancer treatment. This review aims to deliver a comprehensive overview of the glycosylation, function, and research progress of MUC21 in multiple types of cancer and infectious diseases.

## 1 Introduction

Mucins are a class of highly glycosylated large proteins composed of a series of tandem repeat sequences (TR), including proline, serine, and threonine residues (1). These sequences form a central region, and on the epithelial cells, they create a protective barrier (2). Mucins are divided into secreted and transmembrane types. Secreted mucins include MUC2, MUC5AC, MUC5B, MUC6, MUC7, MUC8, MUC9, and MUC19, which can exist in gelation and non-gelation forms (3). Transmembrane mucins consist of a transmembrane domain and a cytoplasmic domain, including MUC1, MUC3A, MUC3B, MUC4, MUC12, MUC13, MUC14, MUC15, MUC16, MUC17, MUC20, MUC21 (Epiglycanin), and MUC22 (4, 5). The MUC family, not only being a critical component in defending against pathogenic microorganisms, is also perceived as a promising target for human tumor diagnosis, treatment, and prognosis.

Mucins are the main components of the mucus layer, playing a role in the body’s anti-infective mechanism (6). In the development of infectious diseases, mucins play a crucial role, and are regulated by cytokines (7). As a transmembrane mucin, the expression of MUC21 is correlated with periodontal inflammation, gingival Amebic infection, Type 1 diabetes, RSV (respiratory syncytial virus) invasion, Mycoplasma pneumoniae pneumonia infection, and the severity of COVID-19 (8–13). The molecular properties of mucins are extremely complex, and extensive research is still needed to fully understand their role in maintaining mucus and resisting infections.

Mucins have garnered significant attention in tumor diagnosis and treatment, with some such as MUC1 and MUC16 being extensively utilized in the research of blood serum diagnostic markers (14, 15). MUC1, the initial mucin to be recognized, has been linked to pancreatic cancer upon its discovery (16). Its presence has been observed in the majority of epithelial cells (17). Additionally, studies have shown that MUC1 is elevated in different types of tumor tissues including breast cancer, ovarian cancer, lung cancer, and colorectal cancer (14). MUC16 (CA125) is the largest transmembrane mucin, which is overexpressed on the surface of ovarian cancer cells and enters the bloodstream, it has become one of the recognized serum biomarkers for ovarian cancer (15). MUC16 is also a significant and promising biomarker for the diagnosis and progression of lung cancer, with the level of its elevation detectable in the blood, which can aid in early diagnosis of pancreatic cancer (18, 19). Here, we introduce a new mucin MUC21, which is the murine mucin’s human homolog (1). Recent research has found that the MUC21 glycoform can differentiate between esophageal squamous epithelial cells and esophageal squamous cell carcinoma (20), and is downregulated in laryngeal squamous cell carcinoma (21). It also closely correlates with the development of lung adenocarcinoma with EGFR mutations (22). MUC21 is a unique immunohistochemical biomarker that distinguishes between lung adenocarcinomas and epithelial mesotheliomas (23). In addition to inducing glioblastoma survival and migration through STA/AKT pathway, MUC21 also regulates SLITRK5 gene expression, thereby controlling Hedgehog signaling pathway to promote melanoma progression (24, 25). MUC21 can also be used as a specific phagocytosis regulatory factor to predict the recurrence and efficacy of thyroid cancer (26). In summary, MUC21 exhibits specific expression in tumor types such as lung adenocarcinoma, melanoma, and glioblastoma, making it a potential candidate for the diagnosis, treatment, and prognosis of these tumors.

## 2 Simple description of MUC21

In 1975, Epiglycanin, a type of mucin, was first reported to be expressed on the surface of glycoproteins in highly malignant TA 3-Ha cells (27, 28). TA 3-Ha originated from a spontaneous tumor in A/HeHa mice in 1949, and is a variant of TA 3 cells (29). In recent years, Itoh and his colleagues discovered a new transmembrane mucin, which was identified as the human counterpart of mouse Mucin 21 (Epiglycanin), and was named MUC21 (1). Mucin, as an epithelial defense molecule, is commonly used as a target for tumor diagnosis and treatment in clinical settings (2). Additionally, there have been observations indicating that mucin expression and glycosylation undergo changes in the presence of infections and other diseases (30).

### 2.1 The structure of MUC21

This new mucin’s MUC21 gene is located in the human MHC I region (1). MUC21 is a high-molecular-weight glycoprotein that possesses transmembrane mucin functions. It is composed of a mucin domain, which consists of 28 different tandem repeat sequences (each repeat sequence corresponds to a serine/threonine-rich region), a stem domain, a transmembrane domain, and a cytoplasmic tail (1).

### 2.2 The MUC21 glycoform

Mucins are present on the outer layer of epithelial cells and are thought to have a crucial role in protecting epithelial cells (6). In addition, they are involved in a range of processes, such as the renewal of epithelial cells, the differentiation of cells, adherence of cells, and the transportation of substances to mucous epithelial cells (2, 31). O-glycosylation is an important post-translational modification that influences the biophysical, functional, and biochemical properties of glycoproteins (31, 32). In particular, the mucin-type O-glycans have several cancer-associated structures, including the T and Tn antigens, and sialylated T antigens. The presence of tn and T antigens is associated with the aggressiveness of cancer cells (33, 34). When the biosynthesis of glycans on the MUC21 stops, the formed MUC21 may have a lower degree of glycosylation, and these structural changes could potentially alter the adhesion, invasiveness, and metastatic properties of the cell (35). Therefore, by delving deeper into the mechanisms and functions of glycosylation, researchers can uncover new ways to target glycosylation-related diseases and develop novel therapeutic interventions.

**Figure 1:**
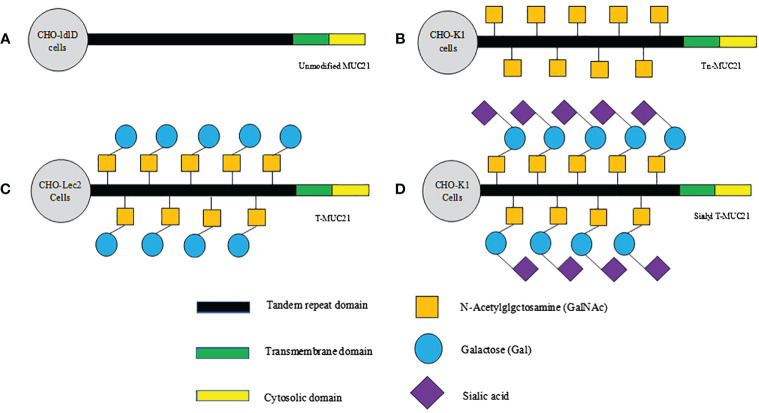
Schematic diagram of hypothesized glycosylation of MUC21 expressed in CHO-ldlD, CHO-Lec2, and CHO-K1 cells. **(A)** Unmodified MUC21 core peptides are expected to be transduced in CHO-ldlD cells, which lack the ability to produce Gal and GalNAc. **(B)** Non-glycosylated Tn-MUC21 is expected to be produced in CHO-ldlD cells (MUC21 containing GalNAc residues is co-cultured with GalNAc). **(C)** T-MUC21, composed of GalNAc and Gal, is expected to be produced in CHO-Lec2 cells (CHO-Lec2 cells lack the ability to prolong sialic acid along their carbohydrate chain). **(D)** Sialyl T-MUC21, composed of GalNAc, Gal and sialic acid, is expected to be produced in CHO-K1 cells.

The recent pathological studies rely on monoclonal antibodies targeting the specific glycoform of human MUC21, providing evidence for the hypothesis that glycoform play a significant role in determining malignant behavior (36). Tian et al. utilized three antibodies to identify the different epitopes of MUC21. One of them was a polyclonal antibody targeting the 20 amino acids in the cytoplasmic tail, while the other two were monoclonal antibodies (mAbheM21C and mAbheM21D) that bound to MUC21 with different glycosylation forms (20). mAbheM21C binds to Tn-MUC21, T-MUC21, and sialylated T-MUC21, while mAbheM21D binds to unmodified MUC21 and Tn-MUC21 core peptides (20)(**Figure 1**). Further investigation into the recognition and unique functions of MUC21 glycoform may provide novel strategies for tumor diagnosis and treatment.

## 3 MUC21-specific glycoform tumor correlations

Specific glycosylation of MUC21 has been implicated in the development of multiple tumors. For example, differentiated and undifferentiated esophageal epithelial cells can be distinguished based on the glycosylation type of MUC21 (20). The latest research indicates that specific glycosylated MUC21 may be involved in the development of lung adenocarcinoma with EGFR mutations (22). In addition, the over-expression of human T-MUC21 and sialylated T-MUC21 confers cells with anti-apoptotic characteristics, whereas unglycosylated MUC21 and Tn-MUC21 do not possess this characteristic (37, 38). These findings underscore the importance of glycosylation in regulating the function of mucins. Moreover, through the development of specific antibodies targeting the MUC21 cytoplasmic tail, researchers have also discovered the expression of MUC21 in the cytoplasm of glioblastoma cells (25). Hence, a mounting array of data suggests that the biological functions of mucins are determined by the glycosylation type, and the development of tools that can accurately and efficiently detect the glycosylation type of mucins and other antigens on tumor cells will aid in treating tumors.

### 3.1 Esophageal squamous cell carcinoma

Research has indicated that the alteration in the MUC21 glycosylation can be used to differentiate between esophageal squamous epithelium and esophageal squamous cell carcinoma (20). MUC21 is expressed in the squamous epithelium of the esophagus, where its O-glycosylation extension form is visible in normal squamous epithelial cells. However, the MUC21 expressed by esophageal squamous carcinoma cells does not attach O-glycosylation (20). Hence, the invasiveness of tumors may be related to the shortening of O-linked polysaccharides (33, 34). The results suggest that the biological traits of MUC21 may depend on its glycosylation (20). In summary, further research on the regulation of the MUC21 glycoform could potentially provide new strategies for the diagnosis and treatment of esophageal squamous cell carcinoma.

### 3.2 Lung adenocarcinoma

Mucins are highly glycosylated proteins, their function is related to glycosylation (39). In normal conditions, mucins function as a protective barrier for lung epithelial cells (2). However, when the regulation is disrupted, these proteins will promote tumor development and metastasis (40). Mucins aid in determining the pathological biological characteristics of tumor cells, such as MUC1 (CA153) and MUC16 (CA125), which are used as biomarkers for monitoring malignant tumors (14, 15). Recent studies have shown that MUC21 is the main cause of the difference between lung adenocarcinoma and lung squamous cell carcinoma (41). Specifically, lung adenocarcinoma exhibits upregulation of genes related to mucin O-chain glycosylation, suggesting that the detection of MUC21 expression may serve as a targeted treatment for lung adenocarcinoma (41).

Recent studies have found that MUC21 is expressed in lung adenocarcinoma (36), and is associated with the invasive behavior of tumor cells (42). Ahmad and colleagues also discovered that MUC21 shows high levels of expression in the micropapillary structures of lung adenocarcinoma, especially during the transition from pure lepidic to micropapillary tumors through the presence of low papillary lepidic lesions (35). This suggests that MUC21 may be associated with the formation of micropapillae structures in lung adenocarcinoma. Further studies are needed to elucidate the specific mechanisms by which MUC21 contributes to the pathogenesis of lung adenocarcinoma and explore its potential as a biomarker for predicting disease progression and treatment response.

Moreover, the development of EGFR-mutated lung adenocarcinomas may also be associated with MUC21 protein, which exhibits specific glycosylation states (35). Among them, the micropapillae structure is crucial for the promotion of tumor development, thus potentially determining the malignancy potential of EGFR-mutated lung adenocarcinomas (43). This is supported by the use of polyclonal antibodies targeting different glycosylation states of MUC21, together with two monoclonal antibodies (heM21D and heM21C) to detect the MUC21 protein, which were named MUC21P, MUC21C, and MUC21D. The polyclonal antibody MUC21P binds to the cytoplasmic tail, monoclonal antibody MUC21C binds to Tn-MUC21, T-MUC21, and sialyl T-MUC21, while monoclonal antibody MUC21D binds to unmodified MUC21 and Tn-MUC21 (20, 35). While MUC21P and MUC21C are expressed in the micropapillae structure or low papillary structure, MUC21D is only expressed in the micropapillae structure (35). This indicates that MUC21D is associated with micropapillae structure. High expression of MUC21D and MUC21P is associated with lymphatic invasion, whereas MUC21D high expression is associated with poor RFS (no recurrence survival) in patients (35). These results imply that frequent lymphatic invasion is the basis of the invasive biological behavior of the micropapillae structure, and the expression of MUC21D is highly correlated with the invasiveness of the micropapillae structure. Therefore, detecting MUC21D may serve as a prognostic indicator for predicting the recurrence of EGFR-mutated lung adenocarcinomas.

## 4 MUC21 regulates cellular functions

MUC21 has been found to have a significant impact on tumor development and is also involved in various essential biological processes. These processes include cell apoptosis, cell adhesion, and cytotoxicity. Additionally, understanding the complex role of MUC21 in these processes can provide valuable insights into underlying mechanisms of disease and potential treatment strategies. Ultimately, further research on MUC21 and its multi-faceted role in cellular functions can lead to advancements in the field of medicine and potentially improve patient outcomes.

### 4.1 The expression of O-glycosylated MUC21 enhances the cell’s anti-apoptotic ability

Increasing evidence suggests that mucin expression is associated with the invasive and metastatic abilities of cancer cells (44). When MUC1 or MUC13 is highly expressed, the anti-apoptotic capacity of cells increases (44, 45). Additionally, previous studies have indicated that epithelial cancer cells with O-glycosylated MUC1 possess resistance to apoptosis (46). Therefore, O-glycosylated MUC21 may also be associated with the cellular anti-apoptotic capacity.

Tian et al. discovered that the anti-apoptotic capability of CHO cells expressing O-glycosylated MUC21 increased, with the expression of T-MUC21 and sialylated T-MUC21 in these cells imparting an anti-apoptotic characteristic (37). Conversely, the expression of unmodified MUC21 or Tn-MUC21 did not endow these cells with an anti-apoptotic trait (37). Considering previous findings, it appears that the TR and intracellular proteins of MUC21 are also necessary conditions for anti-apoptosis, thus, the potential anti-apoptotic mechanism of MUC21 may be related to its specific glycosylation (37).

### 4.2 MUC21 regulates cellular adhesion

In recent years, it has been discovered that cell adhesion plays a crucial role in tumor development and metastasis (47). The anti-adhesive and steric hindrance properties of mucins are vital in many biological processes (39). During the process of tumor development, the reduction in cell adhesion function can lead to an enhancement in cell-to-cell interactions, which is a crucial factor in promoting tumor growth (48). These interactions facilitate the survival and migration of cancer cells into the bloodstream, and the formation of metastases in distant organs from the primary tumor (48). MUC21, the first mucin discovered to be linked to cancer cell advancement, inhibits cell adhesion by creating steric hindrance on the cell surface (39). Yi et al. observed significant morphological changes in cells after MUC21 transfection, identifying a TR structure in MUC21 as mediating the loss of cell adhesion, leading to a loss of cell adhesion ability (39). This demonstrates that, through the highly glycosylated TR domain, MUC21 prevents cell-to-cell and cell-to-matrix interactions, thereby inhibiting cell adhesion and cell-cell, cell-matrix adhesion through steric hindrance (39). Reduced cell adhesion promotes cell invasion and migration functions, thereby leading to tumor metastasis, especially pronounced in lung adenocarcinoma (36).

The micropapillary structure of lung adenocarcinoma is closely associated with cancer cell metastasis, often leading to poor prognosis (49, 50), and it has a strong correlation with lymphatic metastasis, lymphatic invasion, and non-bronchiolar alveolar carcinoma subtypes (51). Miyoshi et al. hypothesized that the reduction in cellular adhesion of micropapillary lung adenocarcinoma could be due to the modulation of lymphatic vessels, which promotes cell invasion and spread, thus affecting tumor metastasis (52, 53). Additionally, evidence suggests that micropapillary-structured cancer cells can disrupt the entire calcium-binding-to-connectin-to-actin network, thereby influencing the disassembly of adhesion junctions and the release of non-adherent invasive and metastatic cells (52). A growing body of research suggests that tumor metastasis is linked to a decrease in cell adhesion (54). In summary, MUC21 expression can affect the formation of micropapillary structures, thereby promoting the development of lung adenocarcinoma.

### 4.3 MUC21 resists cytotoxicity mediated by T cells and NK cells

Immunotherapy is currently a hot topic in cancer treatment research, yet the efficacy of these treatments is far from optimistic due to the cancer cell’s capability to suppress immunosuppression (55). As a result, it is essential to identify these immunosuppressive pathways and create novel approaches to combat the immune system resistance exhibited by cancer patients to immunotherapy. MUC4’s spatial blocking effect aids in the evasion of the immune system’s attack by cancer cells (56). Notably, some studies have demonstrated that on the cell surface, MUC4 can diminish the effectiveness of anti-cancer medications by inhibiting the identification of targeted cells (57, 58). Furthermore, the MUC21 on the surface of tumor cells inhibits the interaction between cytotoxic T cells and MHC molecules on tumor cells (59, 60). Moreover, MUC21 purified from TA3-Ha cells can also produce immunosuppressive effects *in vivo (* 42). In summary, MUC21 can serve as an effective immunosuppressant, inhibiting CAR-T cell and CAR-NK cell anti-tumor functions, thereby protecting the cancer cells from attack by NK and CD8^+^T cells (61). This is accomplished through the generation of spatial blocking, hindering the interaction between immune cells and cancer cells, which plays a crucial role in immune system escape (61). This indicates that blocking MUC21 could be a valuable approach to improve cancer immunotherapy.

## 5 The mechanism of tumor development related to MUC21 and its clinical implications

The family of mucins plays a role in multiple stages of tumor growth, such as cell adhesion, transition between epithelial and mesenchymal states, signaling within cells, and the creation of the tumor microenvironment (62). Metastasis of tumors depends on the anomalous activation of various pathways within tumor cells and the creation of intricate microenvironments. These microenvironments play a role in facilitating the invasion and spread of tumor cells (63). The precise mechanisms by which mucins contribute to tumor invasion and metastasis are still being studied, but it is clear that they play a crucial role in shaping the local tumor microenvironment (2). Among them, mucin family member MUC21 is highly expressed in glioblastoma, thyroid cancer, melanoma, and lung adenocarcinoma, and plays a significant role in their development. Numerous research results indicate that MUC21 has the potential to serve as a blood serum biomarker and therapeutic target. In summary, understanding the role of mucins in cancer progression may lead to the development of targeted therapies aimed at disrupting these interactions and inhibiting tumor growth and metastasis (**Table 1**).

**Table 1:** MUC21 expression related tumors.

Tumor type	MUC21 expression degree	Function
Lung adenocarcinoma	High expression	Participate in the formation of micropapilla structure, decrease cell adhesion and promote tumor metastasis.
Thyroid carcinoma	High expression	It was significantly related to the recurrence-free survival of thyroid cancer.
Glioblastoma	High expression	Regulation of STA/AKT pathway induces survival and migration of glioblastoma.
Melanoma	High expression	Regulate SLITRK5 and Hedgehog signaling pathways to control the development of melanoma.
Epithelioid mesothelioma	Low expression	It is used to differentiate from lung adenocarcinoma.
Laryngeal squamous cell carcinoma	Low expression	It may be related to the development of laryngeal squamous cell carcinoma.
Esophageal squamous cell carcinoma	Non-O-glycosylation MUC21	The shortening or absence of O-glycans in MUC21 is often associated with the aggressiveness of cancer.

### 5.1 Glioblastoma

Wang et al. discovered that MUC21 is aberrantly overexpressed in human glioblastoma (GBM) tissues and cell lines, and correlates with the clinical and pathological features as well as tumor recurrence in GBM patients (24). Their research also indicated that *in vitro*, MUC21 has the ability to enhance the activity and movement of GBM cells, as well as stimulate tumor growth *in vivo (* 24). The migration and invasion of GBM cells could be affected by MUC21 through its impact on cell adhesion in this research (24). They indicated that MUC21 may inhibit cell-cell extracellular matrix interactions and interfere with cell-cell adhesion, thus suppressing cell-cell adhesion molecules and surface integrins, and ultimately impacting tumor metastasis (23, 36, 53). Further research also found that MUC21 can promote the development of GBM through the STAT3/AKT pathway, providing a basis for MUC21 to serve as a potential serum biomarker and therapeutic target for GBM patients (24). By targeting MUC21 or its downstream signaling pathways, it possible to inhibit the spread of GBM and improve patient outcomes. Overall, this study provides valuable insights into the role of MUC21 in GBM progression and highlights its potential as a therapeutic target.

### 5.2 Thyroid cancer and melanoma

Recent research has revealed a noteworthy increase in the expression of MUC21 in thyroid cancer tissues (26). Furthermore, the expression of MUC21 is significantly linked to the outlook of individuals with thyroid cancer, identified as a potential marker for predicting prognosis (26). Previous studies have shown that the presence of MUC21 is elevated in metastatic melanoma tissues, and individuals with high levels of MUC21 tend to have poorer overall survival rates (25). Furthermore, the study has confirmed that overexpressed MUC21 significantly inhibits the function of SLITRK5, thereby activating the downstream Hedgehog signaling pathway (25). Among them, SLITRK5 is identified as a key player in inhibiting the Hedgehog signaling pathway, which plays a critical role in controlling cell growth and specialization (64). The overexpression of SLITRK5 has been shown to enhance the growth, movement, and invasion of melanoma cells (25). This suggests that SLITRK5 may play a significant role in the development and progression of melanoma, a type of skin cancer. Additionally, by targeting SLITRK5 or modulating its activity, researchers may be able to develop new therapeutic strategies for treating melanoma and potentially other cancers that involve dysregulation of the Hedgehog signaling pathway. While the exact functioning of MUC21 is not yet fully understood, it is essential in the progression of thyroid cancer and melanoma and can serve as a significant prognostic indicator.

### 5.3 MUC21 can serve as a negative biomarker

Currently, the most optimal diagnostic methods for distinguishing between epithelial mesothelioma and lung adenocarcinoma are still under investigation. There is no absolute immunohistochemical marker that can be used for differential diagnosis of epithelial mesothelioma (65). Due to the clinical growth patterns and histological similarities between lung adenocarcinoma and epithelial mesothelioma, it is often misdiagnosed as epithelial mesothelioma. Recently, a discovery was made highlighting the potential of MUC21 as a new negative marker for distinguishing between mesothelioma and lung adenocarcinoma. It was found that the expression of MUC21 was significantly different in lung adenocarcinoma and epithelial mesothelioma. The expression of MUC21 is low in epithelial mesothelioma, but high in lung adenocarcinoma (23). This finding suggests that MUC21 could be a valuable tool in accurately diagnosing these two types of cancer, potentially leading to better treatment outcomes for patients. Further research and validation of MUC21 as a diagnostic marker could greatly impact the field of oncology by improving the accuracy and efficiency of cancer diagnosis. In conclusion, the discovery of MUC21 as a negative marker for mesothelioma and lung adenocarcinoma represents a significant advancement in the field of cancer research and holds promise for improving patient outcomes in the future.

## 6 The antimicrobial activity of MUC21

Mucins play a significant role in the development of infectious diseases. Over-expression of transmembrane mucins can disrupt cellular polarity and intercellular interactions, affecting the integrity of the epithelial barrier (7). During the microbial infection process, mucins in the mucus layer play an important role in host immunity (66). Not only are mucus layers physical barriers, but they are also composed of complex molecular systems with significant immunomodulatory and antibacterial properties (67). Furthermore, bio-enzymes may weaken the mucin’s glycan barrier, allowing bacteria to invade mucosal tissues (68). Studies have shown that the novel transmembrane mucin MUC21 is involved in the defense against infection by pathogenic microorganisms. Mucus and mucins play a significant role in protecting the body from invasion, and the specific defense mechanisms are not yet fully understood.

### 6.1 Anti-infective effect of MUC21 in oral cavity

The first line of defense of the mucosal epithelium is the mucin barrier (6). Mucin serves as both a medium for the growth and food source of commensal bacteria, in addition to binding to invading microbes (69). MUC21, a member of the mucin family, aids in protecting epithelial tissues from pathogen invasion and also protects the host’s microbiome (6). A whole-genome expression profiling study revealed that the expression level of MUC21 in healing gingival tissues significantly increased. Moreover, the expression levels of epithelial and connective tissues also enhanced during the healing process, suggesting that this transmembrane mucin is associated with the healing of oral mucosa (70). RSPO4 has been identified as a potential risk gene associated with periodontitis. Scientific studies have demonstrated that upregulation of RSPO4 results in a notable enhancement in the levels of MUC21 expression, thereby impacting the expression of genes associated with host defense and the integrity of barriers (8). Moreover, when gingival epithelial cells are infected with the parasite Trichomonas gingivalis, MUC21 is the mucin gene that is upregulated most significantly (9). In summary, it was observed that the expression of MUC21 significantly increased in oral epithelial infection or damage, providing evidence for its important role in maintaining the integrity of oral barriers. This indicates that changes in the MUC21 gene are closely related to pathogen infection, and its expression has certain protective effects on the epithelial cells.

### 6.2 Anti-infective effect of MUC21 in intestinal tract

The mucus layer plays a vital role in the intestinal barrier by inhibiting bacterial passage and safeguarding the mucosa of the intestines (10). The mucus layer is not only a physical barrier, but also composed of a complex molecular system, which have important immunomodulatory and antibacterial activities, such as mucin and antimicrobial peptides (67, 71). Transmembrane mucins, such as MUC21, can serve as a source of energy for commensal bacteria, playing a crucial role in the regulation of specific microbial species in the mucus layer (69). Research has indicated that patients with type 1 diabetes experience physical and biological alterations in their intestinal barrier. These changes are linked to disruptions in bacterial communities responsible for regulating mucus and imbalances in the immune system (10). Loss of intestinal barrier integrity causes the symbiotic gut microbiota to activate islet reactive T cells and contributes to the development of autoimmune diabetes (72, 73). Therefore, the integrity of the intestinal barrier and the function of the mucus layer are closely related to the pathogenesis of human pancreatic autoimmunity, which causes disease (10). In summary, by protecting the integrity of the intestinal barrier through regulation of mucins, maintaining the stability of the microbial and immune systems, a new direction for the research of autoimmune diseases has emerged.

### 6.3 Anti-infective effect of MUC21 in respiratory tract

The respiratory tract’s mucosal surface is primarily composed of mucus, which is primarily composed of mucins (74). The primary roles of mucus are to shield the epithelial surface from harm and promote the clearance of substances entering the lungs (74). When diseases occur, abnormal expression of mucins can lead to dysregulation of the mucus barrier function, ultimately resulting in pathological changes (6). It has been reported that respiratory syncytial virus invasion can strongly induce upregulation of MUC21 (11). Additionally, MUC21 is not only among the highly elevated mRNAs in bronchoalveolar lavage fluid obtained from children with severe atypical pneumonia induced by Mycoplasma pneumoniae (12), but also the level of MUC21 mRNA expression is strongly correlated with the severity of COVID-19 (13). From the above research findings, it can be concluded that changes in MUC21 expression are highly correlated with the onset, progression, and prognosis of respiratory tract infections and other diseases.

## 7 Summary and prospects

This paper reviews the expression, structure, and biological functions of MUC21 in various complex tumor types, as well as its role in infectious diseases. MUC21, a glycoprotein with high molecular weight, serves as a transmembrane mucin found on the exterior of healthy cells, playing a crucial protective function. Although the overexpression of MUC21 can improve the mucosal barrier and protective function, thus preventing infection, it often occurs abnormal expression in the development of several malignant tumors, and plays an important role in the development and metastasis of tumors. Research has shown that within the MUC21 domain, there exists a TR domain that has the ability to impede cell-to-cell and cell-to-matrix adhesion by altering TR-dependent steric hindrance. This ultimately boosts the invasive and metastatic capabilities of cancer cells (39, 48). Moreover, a number of studies have shown that MUC21 is involved in the invasion and development of malignant tumors such as lung adenocarcinoma, melanoma and glioblastoma, and it has become a promising therapeutic target for the development of targeted therapies.

The advancement of antibody engineering, ADCs, CAR-T cells, CRISPR-Cas9 technology, and genetically engineered mouse models has revolutionized the landscape of targeted therapy (75). By providing new tools and approaches, these advancements are driving progress towards more effective and personalized treatment options. The continued exploration and utilization of these technologies are crucial for advancing the field of medicine and ultimately improving patient outcomes. For example, the presence of MUC21 glycoform shows a significant correlation with tumor growth. As a result, creating antibodies that target the specific mucin glycoform found in tumor antigens can greatly improve cancer therapy. Secondly, it is found that ADCs method can be used to clear the cancer cells expressing MUC21, which may be a promising targeted therapy (61). Moreover, it is a good method to treat tumor by regulating the downstream signal molecules of MUC21. Unfortunately, we know very little about the downstream signal molecules and related signal pathways of MUC21. Further, immunotherapy plays a crucial role in addressing the elevated rate of tumor recurrence in clinical practice. Research on utilizing anti-MUC21 antibody in immunotherapy is anticipated to shed light on the practical significance of MUC21 in managing diseases. This could lead to significant advancements in medical treatments and potentially improve patient outcomes. Ultimately, the implications of this study could have far-reaching effects on the field of medicine and offer hope for more effective treatments in the future. In addition, the research and application of MUC21 serum detection can provide great convenience for the diagnosis, treatment and prognosis of tumors. Therefore, collaborative endeavors are required to create enhanced targeting agents and appropriate animal models for assessing MUC21-focused treatments (75).

In summary, MUC21 is likely to possess the capability to enhance the migration, invasion and metastasis of multiple cancer cells. This suggests that MUC21 may play a crucial role in the progression of tumors, offering valuable insights into its specific functions in the field of tumor biology. Clarifying the roles of MUC21 in these aspects will contribute to the diagnosis, treatment, and prognosis of tumors. Moreover, due to our limited knowledge of the antibacterial mechanisms of MUC21, this presents a significant challenge for our future research.

## Author contributions

ML: Writing – original draft, Writing – review & editing. HL: Writing – review & editing, Writing – original draft. TY: Writing – review & editing. ZL: Writing – review & editing. YKL: Writing – review & editing. YT: Writing – review & editing. YZL: Writing – review & editing.
